# Fostering the exchange of real-life data across different countries to answer primary care research questions: a protocol for an UNLOCK study from the IPCRG

**DOI:** 10.1038/npjpcrm.2016.48

**Published:** 2016-09-22

**Authors:** Liza Cragg, Siân Williams, Thys van der Molen, Mike Thomas, Jaime Correia de Sousa, Niels H Chavannes

**Affiliations:** 1 International Primary Care Respiratory Group, Edinburgh, UK; 2 Department of General Practice, University Medical Center Groningen, University of Groningen, Groningen, The Netherlands; 3 Faculty of Medicine, Primary Care and Population Sciences, University of Southampton, Southampton, UK; 4 Life and Health Sciences Research Institute, School of Health Sciences, University of Minho, Braga, Portugal; 5 Department of Public Health and Primary Care, Leiden University Medical Centre, Leiden, The Netherlands

## Background

This protocol describes a study that will explore the lessons of UNLOCK (Uncovering and Noting Long-term COPD and asthma to enhance Knowledge) over the past 5 years of sharing real-life primary care data from different countries to answer research questions on the diagnosis and management of chronic respiratory diseases.

UNLOCK is an international collaboration between primary care researchers and practitioners to coordinate and share data sets of relevant diagnostic and follow-up variables for chronic obstructive pulmonary disease (COPD) and asthma management in primary care. It was set up by members of the International Primary Care Respiratory Group (IPCRG) in response to the identified research need for research in primary care, which recruits patients representative of primary care populations, evaluates interventions realistically delivered within primary care and draws conclusions that will be meaningful to professionals working within primary care.^1,2^

The UNLOCK protocol summary was published in the Primary Care Respiratory Journal in 2010.^3^ The primary purpose of UNLOCK is to enable the validation of policy and treatment decisions by using data from unselected primary care populations from diverse contexts in very different countries to evaluate the burden of disease (symptoms, limitations and exacerbations), the natural history of disease, treatment and follow-up and co-morbidities. The unique value of UNLOCK is that data are drawn from primary care databases so there is the potential for longitudinal and cross-sectional studies.

It has been 5 years since the UNLOCK collaboration began. In that time its membership has expanded to include 15 countries: Sweden, Spain, Ukraine, Canada, Greece, UK, Netherlands, Norway, Australia, Portugal, Belgium, India, Germany, Uganda and Chile. UNLOCK Group members now offer access to a range of data sets including big data, such as routine healthcare data covering millions of patients, and smaller data sets collected for specific research purposes.

Individual members of the UNLOCK Group continue to show they value the collaboration through their active participation in twice-yearly meetings, collaboration on studies and the development of new data sets. However, a range of practical issues have hampered the UNLOCK Group’s ability to translate research ideas into studies published in peer-reviewed publications. These include structural challenges in working on a single study with several researchers from different countries, such as cultural differences; different language competencies and comfort in discussing in English; variations in how COPD and asthma diagnosis and management is reimbursed and incentivised in primary care; the difficulties of participants combining a busy demand-led primary care job with research, which is often unpaid and unsupervised; and differences in primary care research infrastructure and the value/credibility accorded to primary care research.

Constraints in working with data sets from different countries and collected for different purposes have also emerged. These include the following:

- Different ethical and data protection requirements across different countries.
- The highly variable size of data sets: 100 to >1,000,000 subjects.
- Differences in data variables and their definitions collected by different countries.
- Differences in coding systems and practices.
- The absence of key variables in some data sets.

This study will analyse and share learning from the first 5 years of the UNLOCK collaboration.

## Aims

The objectives of this study are as follows:

1. To describe and classify the successes and motivation of members of the UNLOCK Group in seeking to share real-life data across regional and country borders to answer research questions and how these have been captured/built upon;
2. To describe and classify the constraints experienced by members of the UNLOCK Group in seeking to share real-life data across regional and country borders to answer research questions and how these have been overcome;
3. To identify methods to improve the effectiveness of future studies involving data sharing across countries to inform future research collaborations;
4. To describe other impacts of UNLOCK, including the development of electronic recording systems, data validation and data extraction.

The study will answer the following research questions:

- What are the incentives, motivations and enablers for sharing real-life data across regional and country borders?
- What are the challenges and constraints for sharing real-life data across borders to answer research questions in the experience of the UNLOCK Group?
- What strategies have been developed to overcome these and how effective have they been?
- What lessons can be drawn about how to improve the effectiveness of future studies involving data sharing across borders?
- Are there any specific challenges to fostering the exchange of primary care data in comparison with other data sets such as public health, prescribing or hospital data?
- What are the core respiratory data variables that should be collected nationally to enable chronic respiratory disease research to better answer primary care research questions?

## Methods

### Design

The study will use the following methodology:

- A document review of material related to the UNLOCK Group using content analysis and thematic analysis to explore the background, expectations, participation of members, development of study ideas, planning of studies and progress reporting at meetings compared with completion of studies. Documents to be reviewed include the following: the UNLOCK protocol; minutes of UNLOCK Group meetings; minutes of UNLOCK Steering Committee meetings; study protocols; draft and published UNLOCK studies; feedback from peer reviewers.
- A structured online questionnaire for UNLOCK Group members to explore their perceptions of the strengths of the UNLOCK Group, the barriers to participating in UNLOCK studies, how these can be overcome and the learning to date.
- Structured interviews with UNLOCK Group members who have acted as lead authors for UNLOCK studies to explore the barriers to undertaking and completing UNLOCK studies, how these can be overcome and the learning to date.
- A review of the data sets held by current UNLOCK members, how these have changed over time and how many of these data sets have been used for UNLOCK studies.

### Analysis

Results from the literature review and the review of UNLOCK documents will be coded thematically. The results of the online questionnaire will be analysed by scoring participants’ prioritisation of key themes. The results of the structured interviews will be analysed by scoring participants’ prioritisation of issues suggested during questions. Responses to a smaller number of open questions will be coded and thematically analysed.

### Reporting

Reporting of findings will use Consolidated criteria for reporting qualitative studies (COREQ).^4^ These standards fit within the suite of EQUATOR reporting guidelines.

## Discussion

This study will explore the UNLOCK Group’s experience of sharing data from routine sources and research data sets. This is particularly timely, as there is growing awareness among healthcare planners, providers and researchers of the potential to make better use of routinely collected health data to develop population medicine approaches including disease prevention, as well as targeted interventions such as risk stratification and personalised prescribing to improve outcomes. Research funders have recently prioritised the challenge of finding ways to break down data silos and translate data into actionable information.^5^

At the same time, there is increased emphasis on the need for research data to be shared. In particular, the sharing of data offers benefits including more efficient and faster progress in improving health, better value for money and higher-quality science. However, challenges in building the culture and resources needed to support data sharing are considerable and include concerns that researchers in resource-poor settings will lose out to better-resourced researchers, fears that increased data sharing will create unacceptable risks for patients and the costs in terms of money and time of data sharing.^6^

The study will focus on data generated by primary care clinicians and researchers to answer research questions of relevance to primary care. There is consensus in policy documents and research agendas that primary care has a key role in the fight against NCDs.^7–10^ Consequently, research needs to reflect primary care priorities, including the need to improve evidence to underpin the primary care approach to diagnosis and assessment and broad management strategies.^2^

Furthermore, there is growing consensus that additional evidence is needed about the process of translating basic scientific discoveries into clinical practice, known as the ‘bench to bedside continuum’. The proposed study will contribute to this evidence by identifying successes, enablers, constraints, challenges and mechanisms to overcome these challenges experienced by the UNLOCK Group as researchers and primary care practitioners collaborating to answer primary care research questions. The study will systematically capture and analyse this learning through an implementation science approach. Implementation science can be defined as follows:

*The scientific study of methods to promote the systematic uptake of clinical research findings and other evidence-based practices into routine practice, and hence to improve the quality and effectiveness of healthcare. It includes the study of influences on healthcare professional and organisational behaviour.^11^*

The experience the UNLOCK Group has developed in exploring and seeking to overcome constraints and structural challenges offers valuable learning for other researchers seeking to share and to analyse data sets from different countries to answer questions about the needs of primary care populations. The study is expected to identify recommendations on study design and implementation, including ensuring optimum data variables, primary care coding options, use of data from multiple countries, use of data sets collected for different purposes and ways to support primary care clinicians to participate in research. Findings are also likely to be applicable to other disciplines.

## Funding

The IPCRG provided funding for this research project as an UNLOCK Group study for which the funding was obtained through an unrestricted grant by Novartis AG, Basel, Switzerland. Novartis has no role in study design, data collection and analysis, decision to publish or preparation of the manuscript.
